# Anabolic Hormones Deficiencies in Heart Failure With Preserved Ejection Fraction: Prevalence and Impact on Antioxidants Levels and Myocardial Dysfunction

**DOI:** 10.3389/fendo.2020.00281

**Published:** 2020-05-12

**Authors:** Carmine Bruno, Andrea Silvestrini, Rodolfo Calarco, Angela M. R. Favuzzi, Edoardo Vergani, Maria Anna Nicolazzi, Claudia d'Abate, Elisabetta Meucci, Alvaro Mordente, Raffaele Landolfi, Antonio Mancini

**Affiliations:** ^1^Dipartimento di Medicina e Chirurgia Traslazionale, Università Cattolica del Sacro Cuore, Rome, Italy; ^2^Fondazione Policlinico Universitario a Gemelli IRCCS, Rome, Italy; ^3^Dipartimento di Scienze Biotecnologiche di Base, Cliniche Intensivologiche e Perioperatorie, Università Cattolica del Sacro Cuore, Rome, Italy

**Keywords:** heart failure, cardiovascular endocrinology, antioxidants, hormones, myocardial dysfunctions

## Abstract

**Purpose:** In heart failure with reduced ejection fraction, catabolic mechanisms have a strong negative impact on mortality and morbidity. The relationship between anabolic hormonal deficiency and heart failure with preserved ejection fraction (HFpEF) has still been poorly investigated. On the other hand, oxidative stress is recognized as a player in the pathogenesis of HFpEF. Therefore, we performed a cohort study in HFpEF aimed to (1) define the multi-hormonal deficiency prevalence in HFpEF patients; (2) investigate the relationships between hormonal deficiencies and echocardiographic indexes; (3) explore the modulatory activity of anabolic hormones on antioxidant systems.

**Methods:** 84 patients with diagnosis of HFpEF were enrolled in the study. Plasma levels of N-terminal pro-brain natriuretic peptide, fasting glucose, insulin, lipid pattern, insulin-like growth factor-1, dehydroepiandrosterone-sulfate (DHEA-S), total testosterone (T, only in male subjects) were evaluated. Hormonal deficiencies were defined according to T.O.S.C.A. multi-centric study, as previously published. An echocardiographic evaluation was performed. Plasma total antioxidant capacity (TAC) was measured using the system metmyoglobin –H_2_O_2_ and the chromogen ABTS, whose radical form is spectroscopically revealed; latency time (LAG) in the appearance of ABTS• is proportional to antioxidants in sample.

**Results:** Multiple deficiencies were discovered. DHEA-S deficiency in 87% of patients, IGF-1 in 67% of patients, T in 42%. Patients with DHEA-S deficiency showed lower levels of TAC expressed by LAG (mean ± SEM 91.25 ± 9.34 vs. 75.22 ± 4.38 s; *p* < 0.05). No differences between TAC in patients with or without IGF-1 deficiency were found. A trend toward high level of TAC in patients without hormonal deficiencies compared with patients with one or multiple deficiencies was found. Regarding echocardiographic parameters, Left Atrial and Left Atrial Volume Index were significantly higher in patients with low IGF-1 values (mean ± SD 90.84 ± 3.86 vs. 72.83 ± 3.78 mL; 51.03 ± 2.33 vs. 40.56 ± 2.46 mL/m^2^, respectively; *p* < 0.05).

**Conclusions:** Our study showed high prevalence of anabolic deficiencies in HFpEF. DHEA-S seems to influence antioxidant levels; IGF-1 deficiency was associated with alteration in parameters of myocardial structure and dysfunction. These data suggest a role of anabolic hormones in the complex pathophysiological mechanisms of HFpEF and could represent the basis for longitudinal studies and investigations on possible benefits of replacement therapy.

## Introduction

Heart Failure (HF) is a clinical syndrome characterized by typical signs and symptoms (dyspnea, fluid retention, fatigue, and exercise intolerance) associated with reduced cardiac output and/or elevated intracardiac pressures at rest or during stress. Classically, HF is classified according to left ventricle ejection fraction (LVEF) in two main variants: HF with reduced ejection fraction (HFrEF) characterized by LFEF <40%, and HF with preserved ejection fraction (HFpEF) when LVEF ≥ 50% (1).

These two variants of HF, while sharing common clinical features, have been associated with different pathophysiological models (2) and therapeutic efficacy of medications on mortality (3).

About HFpEF pathophysiology, diastolic dysfunction is the main mechanism involved, but several other alterations, such as left atrial dysfunction, right ventricular dysfunction, pulmonary hypertension, and increased vascular stiffness, have been identified, which contribute to HFpEF (4–7). Advanced age and comorbidities are the leading risk factors for HFpEF. According to a recent proposal, comorbidities play a pivotal role in leading a systemic proinflammatory status that is responsible, via oxidative stress at microvascular level, of functional and structural myocardial alteration typical of HFpEF (8). Among comorbidities, the most important and widely prevalent in HFpEF patients are represented by obesity, diabetes, iron deficiency, chronic obstructive pulmonary syndrome (COPD), hypertension, and renal failure. The clinical course of comorbidities significantly influences HFpEF prognosis (9–11) and many interventions, both pharmacological and non-pharmacological, directed to improve comorbidities have been associated to better clinical outcomes in patients with HFpEF (12).

Regarding HFpEF therapies, many efforts have been made to find effective pharmacological approaches; conventional HF drugs, including ACE inhibitors, sartans, and β1-antagonists (13–15), did not show improvements in HFpEF morbidity and mortality; about mineralocorticoids receptor antagonists (MRI) and neprilisin-inhibitors, *post-hoc* analyses of randomized trials suggest the need of accurate stratification of HFpEF patients, in order to implement “tailor-made” therapeutic strategies (16, 17). In this direction, pharmacogenetics could represent a promising field of investigations (18).

On the other hand, anabolic hormones, insulin-like growth factor-1 (IGF-1), dehydroepiandrosterone-sulfate (DHEA-S), testosterone (T), have an important role on cardiac morphology and function (19–21); Anabolic hormones deficiency, in which the so called “cardiac cachexia” represents the end-stage, demonstrated detrimental impact on disease progression and mortality in heart failure with reduced ejection fraction (HFrEF) (22–24).

The relationship between anabolic hormone deficiency and HFpEF has been poorly investigated. A study of Salzano et al. (25) showed a lower impact of anabolic drive deficiencies in HFpEF than HFrEF, although about half of the HFpEF patients demonstrated single or multiple hormonal deficiency. These data were partially in agreement with our preliminary study on prevalence of anabolic deficiencies in HFpEF (26).

No data were reported about the impact of hormonal deficiencies on oxidative stress parameters; therefore, we performed an observational cross-sectional study to quantify anabolic hormonal deficiency prevalence, and to investigate the relationships between anabolic alterations, echocardiographic parameters, and antioxidants levels, with the aim to gain insight into pathophysiology of HFpEF.

## Patients and Methods

Chronic HFpEF patients with NYHA functional class I–III, admitted to the Department of Internal Medicine of the “Fondazione Policlinico Universitario A. Gemelli IRCCS” between April 2016 and May 2019 were recruited. The HFpEF diagnosis was based on current European Society of Cardiology guidelines (1). Patients with symptoms and signs of HF, elevated natriuretic peptides levels, N-terminal proBNP > 125 pg/mL and LVEF > 50%) and echocardiographic evidence of diastolic dysfunction were considered. The echocardiographic criteria of diastolic dysfunction were defined as left atrial volume index (LAVI) > 34 mL/m^2^, left ventricular mass index left atrial volume index (LAVI) > 34 mL/m or a left ventricular mass index (LVMI) ≥ 115 g/m^2^ for males, and ≥95 g/m^2^ for females. Doppler parameters were a ratio of transmitral early filling velocity to tissue early diastolic mitral annular velocity (E/e′) ≥ 13 and a mean e′ septal and lateral wall <9 cm/s.

Patients with acute HF, NYHA class IV, end stage renal disease, liver cirrhosis, neoplastic or autoimmune diseases were excluded, as well as patients with known endocrinopathies, taking hormonal replacement therapy or previous/current amiodarone treatment.

Information about physiological and medical history, including the main risk factors for cardiovascular disease and pharmacological therapy, were acquired. Standard medical therapy, including loop diuretics, angiotensin converting enzyme inhibitors (ACEi), angiotensin II receptor blockers (ARB), and beta-blockers (BBs) had to be stable for at least 2 months. We investigated the prevalence of the following comorbidities: arterial hypertension, diabetes mellitus (DM), chronic obstructive pulmonary disease (COPD), renal failure, anemia, atrial fibrillation, peripheral artery disease, or coronary artery disease.

Body weight was measured in light clothes with an electronic scale (Seca 910; Seca, Ham- burg, Germany) to the nearest 0.1 kg and height was measured with a stadiometer (Seca 220 tele-scopic measured rod; Seca, Hamburg, Germany) to the nearest 0.1 cm. Body Mass Index (BMI) was calculated with the formula Body weight (kg)/[height (m)]^2^. All patients signed written informed consent, according to the declaration of Helsinki. The study was approved by the Local Ethics Committee.

In all patients, venous blood samples were collected in lithium-heparin tubes in the morning after an overnight fast and after a supine rest of at least 15 min, in order to evaluate plasma levels of N-terminal pro-brain natriuretic peptide (NT-proBNP), plasma fasting glucose, IGF-1, DHEA-S. In male subjects, total testosterone (T) was also assayed. All samples were centrifuged within 2 h after collection and separate plasma aliquots were stored at −80°C until assayed.

A complete echocardiography evaluation was performed (Affiniti 70c, Echocardiography Philips, Philips s.p.a. Milan, Italy), calculating the following parameters: left ventricular ejection fraction (LVEF), left ventricular end-diastolic volume (LVEDV), left ventricular end-systolic volume (LVESV), septal thickness (IVS), posterior wall thickness (PW), peak E-wave velocity (E), peak A-wave velocity (A), E/A ratio, pulsed- wave TDI E′ velocity (E′), E/e′ ratio, deceleration time (DT), left atrial volume (LAV), indexed left atrial volume (LAVI), systolic pulmonary artery pressure (SPAP), tricuspid annular plane systolic excursion (TAPSE), right ventricular mid cavity diameter value (RVEDV), TAPSE/SPAP ratio, and tricuspidal peak velocity (TPV).

NT-proBNP plasma concentrations, DHEA-S, T, and IGF-1 were measured using immunochemiluminometric assays on a Roche Modular E170 analyzer (Roche Diagnostics, Indianapolis, IN, USA). The intra-assay and inter-assay CV for all hormones were, <5.0 and <7.0%, respectively. Echocardiographic and hormonal parameters were obtained in a stable clinical condition.

Our laboratory considered the following ranges as normal: NT-proBNP (>126 pg/mL), DHEA-S (800–3,500 ng/mL), and T (2.5–8.4 ng/mL). Values equal or below the lower normal limit of normal were defined as deficiency. IGF-1 deficiency was defined according to T.O.S.C.A. registry criteria (i.e., 122 ng/mL for age range >55 years, 109 ng/mL for range 55–64 years, 102 ng/mL for range 65–74, 99 ng/mL for age >75 years), referring to the 33th percentile of a male population with Chronic Heart Failure due to age-related variations (27).

Plasma total antioxidant capacity (TAC) was evaluated using the method developed by Rice-Evans and Miller (28). The method is based on inhibition, determined by antioxidants, of the absorbance of the radical cation 2,2′-azinobis (3-ethylbenzothiazoline-6 sulfonate) (ABTS•+) formed by interaction between ABTS (150 μM) and ferrylmyoglobin radical species, generated by activation of metmyoglobin (2.5 μM) with H_2_O_2_ (75 μM). Aliquots of the frozen plasma were thawed at room temperature and 10 μL of the samples were tested immediately. The manual procedure was used with only minor modifications, as previously described (29). The reaction was started directly in cuvette through H_2_O_2_ addition after 1 min equilibration of all other reagents and followed for 10 min, monitoring at 734 nm, typical of the spectroscopically detectable ABTS•+. The presence of chain-breaking antioxidants induces a lag time (the “Lag phase”) in the accumulation of ABTS•+ proportional to antioxidants concentration and expressed as length of such Lag phase (s). This assay mainly measures non-protein and non-enzymatic antioxidants that are primarily extra-cellular chain-breaking antioxidants, such as ascorbate, urate, and glutathione. Trolox, a water-soluble tocopherol analog, was used as a reference standard. Absorbance was measured with a Hewlett-Packard 8450A UV/Vis spectrophotometer (Palo Alto, CA) equipped with a cuvette stirring apparatus and a constant temperature cell holder. Measurements of pH were made with a PHM84 Research pH meter (Radiometer, Copenhagen, Denmark); the electrode response was corrected for temperature. Intraassay CV was <8%.

### Statistical Analysis

Patients with normal values and hormonal deficiencies were identified for each single measured hormonal parameter. Mean and Standard Error of the Mean (SEM) were used to describe quantitative variables, absolute and relative frequencies for qualitative variables. Student *T*-Test was used to evaluate the differences in echocardiographic parameters, NT-proBNP values, BMI, and age between patients divided in groups according to the presence or absence of hormone deficiency (in particular, IGF-1 and testosterone deficiencies).

Mann-Whitney *U*-Test was used to compare the same parameters between patients with or without DHEA-S deficiency. In order to investigate the associations between hormonal deficiency and comorbidities, Fisher's exact or Chi-squared test was used. A value of *p* < 0.05 was considered statistically significant and the analysis was performed using Prism 6.

## Results

Our cohort of patients consisted of 84 patients, 36 men, and 48 women, aged between 59 and 98 years (mean ± SEM: 80.31 ± 0.94 years). Table 1 showed the demographic and clinical features of the entire cohort.

**Table 1:** Demographic, clinical features, and hormonal levels of the cohort.

		**Total population = 84 pts**	**%**	**Mean ± SEM**
Male		36		
Female		48		
Age (years)				80.31 ± 0.94
BMI (kg/m^2^)				27.09 ± 0.53
NYHA	II	27	32.2%	
	III	57	67.8%	
GFR^*^ (mL/min/1.73 mq)				61.00 ± 2.65
NT-PROBNP (ng/mL)				4,376.49 ± 647.34
IGF-1 (ng/mL)				95.98 ± 0.05
DHEA-S (ng/mL)				411.19 ± 38.06
T^**^ (ng/mL)				1.75 ± 0.19

*DHEA-S, dehydroepiandrosterone-sulfate; GFR, glomerular filtration rate; IGF-1, insulin-like growth factor-1; NT-proBNP, N-terminal pro-brain natriuretic peptide; T, total testosterone*.

* *GFR was calculated with MDRD formula*.

** *Evaluated only in male patients*.

Table 2 showed prevalence of comorbidities.

**Table 2:** Prevalence of comorbidities.

**Comorbidities**	***n***	**%**
Hypertension	69	82.1
PAD/CHD	55	65.4
Anemia	57	67.8
COPD	33	39.2
Diabetes	31	36.9
Atrial fibrillation	36	42.8

*COPD, chronic obstructive pulmonary disease; PAD/CHD, peripheral artery disease/coronary heart disease*.

**Figure 1:**
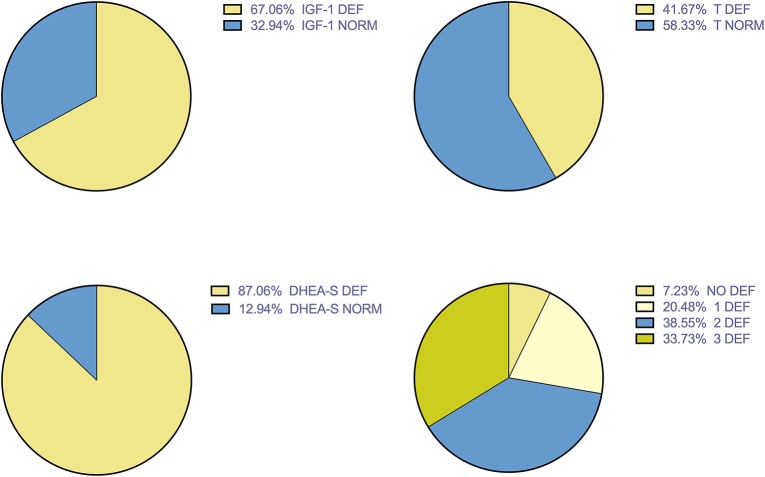
Prevalence of hormonal deficiencies.

Figure 1 showed the prevalence of hormone deficiencies. DHEA-S deficiency represent the most prevalent anabolic hormone deficiency.

**Figure 2:**
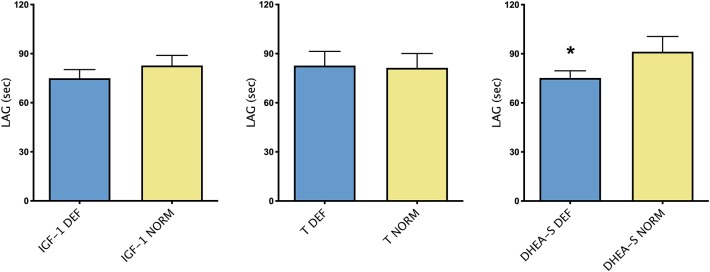
Mean ± SEM of TAC, expressed by LAG (s), in patients divided according to the presence/absence of each hormonal deficiency. *p < 0.05.

Figure 2 showed differences in TAC, expressed by LAG, in group of patients with or without each hormone deficiency. As it could be noticed, higher LAG levels were observed in patients with DHEA-S deficiency compared with patients with normal hormonal values.

**Figure 3:**
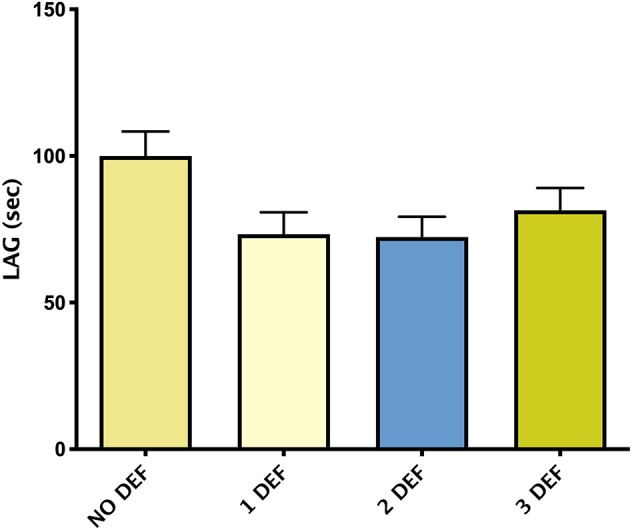
Mean ± SEM of TAC, expressed by LAG (s), in patients divided according to the number of hormonal deficiencies observed.

Figure 3 showed mean ± SEM LAG in patients divided by the number of hormonal deficiencies observed. Although not significant, patients without hormonal deficiencies presented a trend toward higher levels of LAG compared with patients with one or more hormonal deficiencies.

Table 3 showed echocardiographic parameters in patients with or without IGF-1 deficiency. Patients with IGF-1 deficiency showed higher LAV and LAVI. About prevalence of comorbidities, no statistically significant differences were found between the two groups.

**Table 3:** Echocardiographic parameters in patients with or without IGF-1 deficiency.

	**Normal IGF-1**		**IGF-1 Deficiency**	
	**Mean**	**SEM**	**Mean**	**SEM**
Age (years)	80.26	1.48	80.52	0.89
LVEDV (mL)	90.29	3.82	82.88	2.32
LVESV (mL)	39.68	2.12	38.27	1.82
IVS (mm)	13.56	1.05	14.39	0.95
PW (mm)	10.85	0.28	11.00	0.38
E (mm/s)	340.29	54.09	394.51	44.43
A (mm/s)	356.96	59.21	468.26	51.05
Dt (ms)	220.95	15.01	241.76	16.46
EF (%)	58.14	0.74	59.75	2.65
E/A	1.12	0.27	0.88	0.04
E/E′	11.14	0.57	11.84	0.51
LAV (mL)	72.82	3.78	90.84^*^	3.86
LAVI (mL/m^2^)	40.56	2.46	51.03^*^	2.33
TAPSE (mm)	21.31	3.78	21.37	0.51
RVEDV (mm)	28.12	0.77	27.19	0.55
SPAP (mmHg)	34.16	1.55	37.06	1.52
TAPSE/SPAP	0.69	0.05	0.59	0.02

*A, Peak A-wave velocity; DT, Deceleration time; E, Peak E-wave velocity; E′, Pulsed-wave TDI E′ velocity, E/A, E/A ratio, E/E′ ratio; IVS, Septal thickness; LAV, left atrial volume; LAVI, left atrial volume indexed; LVEDV, left ventricular telediastolic volume; LVEF, left ventricular ejection fraction; LVESV, left ventricular telesystolic volume; SPAP, Systolic pulmonary artery pressure; PW, Posterior wall thickness; RVEDV, right ventricular mid cavity diameter value; TAPSE, Tricuspid annular plane systolic excursion; TAPSE/SPAP, TAPSE/SPAP ratio*.

* *p < 0.05*.

No statistically significant differences in echocardiographic parameters and in prevalence of comorbidities were found in patients with or without T and DHEA-S deficiency.

## Discussion

Our data showed high prevalence of anabolic hormones deficiencies in HFpEF. This confirms previous preliminary report of our group (26), underlying DHEA-S deficiency as the most prevalent. They also partially agree with the study of Salzano et al. (25), in which demographic and clinical features of their cohort of patients were slightly different (better NYHA class, lower mean age). However, our data are not simply related to the age of patients since no significant differences were observed between patients with normal or low levels of the studied hormones.

Furthermore, it is matter of discussion if alterations in anabolic drive observed in HF patients represent a cause or a consequence of the clinical syndrome. Our data, showing a reduced TAC in patients with DHEA-S deficiency, are in favor of a role of hormonal deficiency in influencing HFpEF pathogenesis and/or pathophysiology. In fact, over the past several decades, clinical and experimental studies (30–33) have provided substantial evidence that oxidative stress (OS), defined as an excessive production of reactive radical species of oxygen (ROS) compared to antioxidant defenses, is enhanced in heart failure (HF). ROS in the heart are involved in the lowering of contractile function, myocardial growth and hypertrophy, extracellular matrix remodeling, fibroblast proliferation, and definitely in the breakthrough and progression of the disease (34). Mechanisms involved have been previously reviewed (35). Oxidative stress is considered a main player in pathogenesis of HF, but with different pathways in the two models of HF. In fact, as recently proposed, while in HFrEF the process starts with primary ischemic or oxidative damage of cardiomyocytes, in HFpEF a cascade of events is increased by the systemic pro-inflammatory state related to multiple co-morbidities (36). For instance, diabetic patients showed a dysregulation of the innate and adaptive immune systems in myocardial tissue (37, 38). In obesity and insulin resistance visceral adipose tissue shows a higher CD8+:CD4+ T-cell ratio and macrophage M1 polarization (39), that represent a chronic pro-inflammatory response that, via cytokines activation, contributes to HFpEF progression (40). Cardiac macrophages—and related oxidative stress—are responsible of inducing cardiomyocyte apoptosis and interstitial fibrosis in the HFpEF heart (40). Moreover, the resultant endothelial damage leads to microvascular coronary alterations, and, ultimately, to myocardial dysfunction (8).

Finally, a new important field is the differential response to medical therapy, suggested by recent studies about pharmacogenetics, in the view of personalized medicine; a great importance could be exerted by genetic variations of G-protein coupled receptors (GPCRs) which have been studied in some models of heart failure, in particular variants of the β adrenergic receptors have been linked to a worse clinical course of HF (41, 42). Some other classes of drugs could be also indirectly involved, such as polymorphism of AT1R associated with hypertension (43) and dopamine receptor D2 variations linked to obesity (44).

In this complex interplay between inflammation, oxidative stress and individual response to drugs, anabolic hormones impairment could be another mosaic tile. DHEA-S, in fact, is involved in the regulation of OS (45), and also in HF (35). According to literature, DHEA-S can exert reciprocal effects (pro-oxidant or antioxidant) depending on tissue specificity and hormonal levels (46, 47). DHEA-S treatment may reduce lipogenesis and weight gain in rats (48), delay atherosclerosis in rabbits (49), increase insulin secretion and sensitivity in rats (50, 51), and it may reduce cardiac fibrosis in diabetic rats (52). Various investigations showed a potential role of DHEA in antioxidants modulation. Yorek et al. (53) demonstrated that DHEA reduces plasma oxidative stress markers production in arterioles of diabetic rats, such as thiobarbituric acid-reactive substances (TBARS) and superoxide anion, while Aragno et al. (54) observed ROS reduction in hearts of DHEA-treated diabetic rats. It also narrows oxidative stress-induced skeletal muscle damage in diabetic rats (55). *In vivo* and *in vitro* studies showed that DHEA-S counteracts lipid peroxidation (56, 57). Moreover, OS parameters in plasma and in peripheral blood mononuclear cells in diabetic subjects are significantly decreased by DHEA treatment (58).

GH/IGF-1 axis alteration is another factor with high prevalence in our cohort, with an impact on left atrium parameters, considered important indexes of myocardial dysfunction. Although GH is also a regulator of oxidative balance as previously showed, we did not find any influence on TAC in cohort object of our study. It is well-known that IGF-1 is a key factor in cardiomyocytes trophism and contractility. A higher IGFBP/IGF-1 ratio was associated with increased E/e′ ratio, NT-proBNP levels and left atrial enlargement (59). IGF-1 decrease or IGFBP-7 increases were associated with a poor clinical course triggering fibrosis mechanism via increased collagen deposition in the myocardium, one of the mechanisms involved in structural abnormalities leading to diastolic dysfunction. Elevation of soluble suppression of tumorigenicity-2 (sST2), a known collagen synthesis marker from myocardial fibroblast (60) are in favor of this hypothesis. Other links between IGF-1 deficit and diastolic dysfunction were described. Patients with growth hormone and IGF-1 deficiencies exhibit endothelial dysfunction, reduced nitric oxide (NO) production, and high peripheral vascular resistance. GH replacement therapy normalizes NO production and ameliorates peripheral resistance (61). Reduced NO production could contribute to worsening of ventricular compliance in HFpEF patients.

As antioxidants levels are concerned, we observed a trend toward higher levels of TAC in patients with normal hormonal milieu compared with patients with multiple hormonal deficiencies, although not significant. This pattern is not overlapping with one observed in HFrEF as previously described by our group (62); this could reflect a different mechanism underlying oxidative stress related, as above discussed, to a primitive myocardial damage in HFrEF (i.e., ischemic heart disease), and to systemic comorbidities in HFpEF.

Nevertheless, some potential limitations of the present study can be identified. Firstly, the cohort of patients studied although greater than previous reports, needs to be further enlarged. Secondly, TAC as measure of OS should be coupled with other parameters of oxidative damage; finally, our study was focused on IGF-1 levels without evaluating GH secretion via dynamic tests. Therefore, further investigations should be performed to evaluate prevalence of GH deficiency in HFpEF.

## Conclusions

Our study, extending previous observation, confirmed a high prevalence of anabolic hormone deficiencies in patients with HFpEF, with particular relevance of DHEA-S. It seems to be related to antioxidants modulation, with higher levels of TAC in patients without DHEA-S deficiency; considering the role of OS in HFpEF pathogenesis, our data could suggest a pathogenetic involvement of this hormone.

Another anabolic key factor could be IGF-1; patients with low IGF-1levels presented left atrial enlargement that is considered one of main diastolic dysfunction criteria.

In conclusion, HFpEF seems to be a complex model with a reciprocal interaction between comorbidities, including anabolic deficiencies, and myocardial function. Oxidative stress could represent the *trait union* between the three components of the systemic picture, typical of HFpEF.

Therapeutically consequences of these observations remain to be further investigated.

## Data Availability Statement

The datasets generated for this study are available on request to the corresponding author.

## Ethics Statement

The studies involving human participants were reviewed and approved by Ethics commitee of Fondazione Policlinico Universiario A Gemelli IRCSS, Largo A Gemelli 00168, Rome, Italy. The patients/participants provided their written informed consent to participate in this study.

## Author Contributions

CB, AS, AF, and AMa were involved in conception and design of the study. CB, EV, and AS wrote the manuscript. EV, CB, RC, MN, and Cd'A were involved in acquisition and analysis of data. RL, EM, AMa, and AMo were involved in supervision. All authors were involved in critical discussion and approved the final manuscript.

## Conflict of Interest

The authors declare that the research was conducted in the absence of any commercial or financial relationships that could be construed as a potential conflict of interest.
